# Spatial prediction of winter wheat yield gap: agro-climatic model and machine learning approaches

**DOI:** 10.3389/fpls.2023.1309171

**Published:** 2024-01-08

**Authors:** Seyed Rohollah Mousavi, Vahid Alah Jahandideh Mahjenabadi, Bahman Khoshru, Meisam Rezaei

**Affiliations:** ^1^ Soil Science and Engineering Department, Faculty of Agricultural, College of Agriculture & Natural Resources, University of Tehran, Karaj, Iran; ^2^ Soil and Water Research Institute (SWRI), Agricultural Research, Education and Extension Organization (AREEO), Karaj, Iran

**Keywords:** spatial modeling, crop yield, environmental factors, soil properties, learning models, uncertainty analysis

## Abstract

This study aimed to identify the most influential soil and environmental factors for predicting wheat yield (WY) in a part of irrigated croplands in southwest Iran, using the FAO-Agro-Climate method and machine learning algorithms (MLAs). A total of 60 soil samples and wheat grain (1 m × 1 m) in 1200 ha of Pasargad plain were collected and analyzed in the laboratory. Attainable WY was assessed using the FAO method for the area. Pearson correlation analysis was used to select the best set of soil properties for modeling. Topographic attributes and vegetation indices were used as proxies of landscape components and cover crop to map actual WY in the study area. Two well-known MLAs, random forest (RF) and artificial neural networks (ANNs), were utilized to prepare an actual continuous WY map. The k-fold method was used to determine the uncertainty of WY prediction and quantify the quality of prediction accuracy. Results showed that soil organic carbon (SOC) and total nitrogen (TN) had a positive and significant correlation with WY. The SOC, TN, normalized different vegetation index (NDVI), and channel network base level (CHN) were recognized as the most important predictors and justifying more than 50% of actual WY. The ANNs outperformed the RF algorithm with an *R* ^2^ of 0.75, RMSE of 400 (kg ha^−1^), and RPD of 2.79, according to statistical indices. The uncertainty analysis showed that the maximum uncertainty of the prediction map [400 (kg ha^−1^)] was very low compared to the mean value [4937 (kg ha^−1^)] of WY map. Calculation yield gap using the FAO-agro-climatic model showed that the average yield gap of the region was about 50% of actual yield. The findings of this study demonstrated that integrating simulated attainable crop growth using crop model and a set of soil and environmental covariates with the ANNs algorithm can effectively predict WY gaps in large areas with acceptable and reasonable accuracy. The study emphasizes that the implementation of efficient management practices has the potential to enhance agricultural production in the study area and similar regions. These results represent a significant advancement of sustainable agriculture and provide valuable insights for ensuring global food security.

## 1 Introduction

The accurate prediction of wheat yield (WY) is essential for ensuring global food security and supporting sustainable agricultural practices (Ruan et al., 2023). Wheat is one of the most widely cultivated crops worldwide, serving as a staple food for a significant portion of the global population. Therefore, it is imperative to understand the multifaceted factors that influence WY and to develop reliable prediction models. These models can optimize productivity and inform decision-making processes in the agricultural sector (Pant et al., 2021).

Soil and environmental factors play a crucial role in determining WY (Araus et al., 2003; Jahandideh Mahjenabadi et al., 2022). Factors such as soil nutrient content [exchangeable potassium, total nitrogen (TN), etc], pH levels, and organic matter composition affect soil fertility and nutrient availability, ultimately impacting the growth and health of wheat plants (Norouzi et al., 2010; Zhang et al., 2022). In Addition, crop yield is also influenced by a range of factors, including the spatial variability of soil, nutrient availability, landscape characteristics, and management practices. These factors contribute to the genetic potential of the soil-landscape component and the biophysical environment, ultimately affecting crop yield both directly and indirectly (Bobryk et al., 2016). Recent research has highlighted the significant role of field topography in the variation of WY, because it affects soil moisture content and soil properties, which both have a direct impact on crop productivity (Ajami et al., 2020). Various studies have emphasized the importance of integrating terrain attributes with soil and crop variables when modeling yield and soil parameters (Brown et al., 2004; Beaudette et al., 2013).

Traditional WY mapping and measurement are often reliant on intensive field and labor activity, which can be time consuming, expensive, and require the experience of scientists, especially for large areas (Taghizadeh-Mehrjardi et al., 2020; Mousavi et al., 2023). Also, conventional digital soil mapping employs geostatistical approaches that have been widely employed to model the soil or WY relationships between one or several covariates. Kriging model assumes a linear relationship among WY and, covariates, is difficult to model when using a huge number of covariates (Wadoux et al., 2019).

On the other hand, machine learning algorithms (MLAs), as an alternative, have demonstrated great potential for overcoming these limitations (Elavarasan et al., 2018; Wadoux et al., 2019). They have the ability to handle high-dimensional datasets, capture nonlinear relationships, and discern intricate patterns in the data (Van Klompenburg et al., 2020). Over the years, numerous ML algorithms have been employed for crop detection and yield prediction across different locations. By applying these MLAs, researchers have aimed to enhance accuracy and enable informed decision making in agricultural practices (Drummond et al., 2003; Mishra et al., 2016). Among the ML models, random forest (RF) and artificial neural networks (ANNs) have emerged as popular choices for predicting crop yields. RF models leverage an ensemble of decision trees to make robust predictions, while ANNs models simulate the interconnectedness of neurons in the human brain to capture complex relationships (Liu et al., 2012). These algorithms have been successfully applied in various agricultural contexts, such as digital soil mapping (Rostaminia et al., 2021; Mousavi et al., 2022; Khosravani et al., 2023; Rezaei et al., 2023), showcasing their effectiveness in predicting crop yields based on environmental factors and soil properties (Taghizadeh-Mehrjardi et al., 2020; Wang et al., 2020; Basir et al., 2021). As regards, Boori et al. (2023) indicate that the rapid advances in satellite technologies and MLAs, particularly ANNs, have the potential to offer affordable and comprehensive solutions for accurate grain prediction. By utilizing ANNs and other ML models, satellite data can be analyzed to make precise predictions regarding crop yields. A study conducted by Roell et al. (2020) applied the RF model to estimate maps of the winter WY in Denmark by incorporating soil variables, climate factors, and topography attributes. They revealed that the RF model used in the study performed well in predicting WY in the study area. Also, Alvarez (2009) conducted a study focusing on WY prediction in Argentine grassland with the aid of environmental parameters and soil physical properties. The research findings indicated that the ANN outperformed the technique in predicting WY. Similar finding was reported by other researchers, Basir et al. (2021) and Jahandideh Mahjenabadi et al. (2022), which focused on predicting crop yield, that is, wheat and rice by using ANN and RF MLA and found the highly accurate performance for yield prediction.

To meet the growing food demand, global agricultural productivity needs to increase. By 2050, an additional 1 billion tons of cereals will be required, which means increasing production from 2.1 to 3.0 billion tons. This can be achieved by closing the yield gap or increasing the potential yield of crops (Alexandros and Bruinsma, 2012; Hatfield and Beres, 2019). As regards, Fischer et al. (2014) found that increasing potential yield is an important factor in increasing actual yield; therefore, increases in actual yield are a result of improved agronomic practices and would require the implementation of multiple practices. Moreover, when it comes to crop modeling, it is crucial to consider the yield gap calculation as well as the management of nutrients and water supply. By doing so, we can accurately predict crop yields and identify areas where improvements in nutrient and water management can be made to reduce the yield gap and increase productivity.

To the best of our knowledge, there has been limited investigation into modeling actual and gap WY by incorporating the three components of climate, soil, vegetation, and topographic attributes. Jahandideh Mahjenabadi et al. (2022) conducted a study that solely focused on soil biological properties and neglected wheat potential production by crop modeling, or FAO-agro-climatic model, as well as the consideration of yield gap. The impact of environmental covariates and soil physio-chemical properties on the prediction of actual WY was not evaluated. Furthermore, they failed to take into account the uncertainty of the actual WY prediction map, which could be useful in assessing the performance of ML algorithms. As a result, there is still a gap in knowledge regarding the influence of other factors on the actual and potential attainable WY amount in the Pasargad plain. To solve this lack of information and knowledge, this study was conducted with the aim of identifying (i) the primary factors that control actual WY; (ii) investigating the quality of two MLAs, RF, and ANNs in the prediction of actual WY; and (iii) calculating the potential production of WY to achieve the yield gap map in the agricultural lands. The ultimate goal of this research is to prepare a spatial prediction map of the actual, uncertainty, and yield gap of wheat and identify areas with high and low production capability. This information will be useful for future land use planning and agricultural management.

## 2 Materials and methods

### 2.1 Study area description

**Figure 1:**
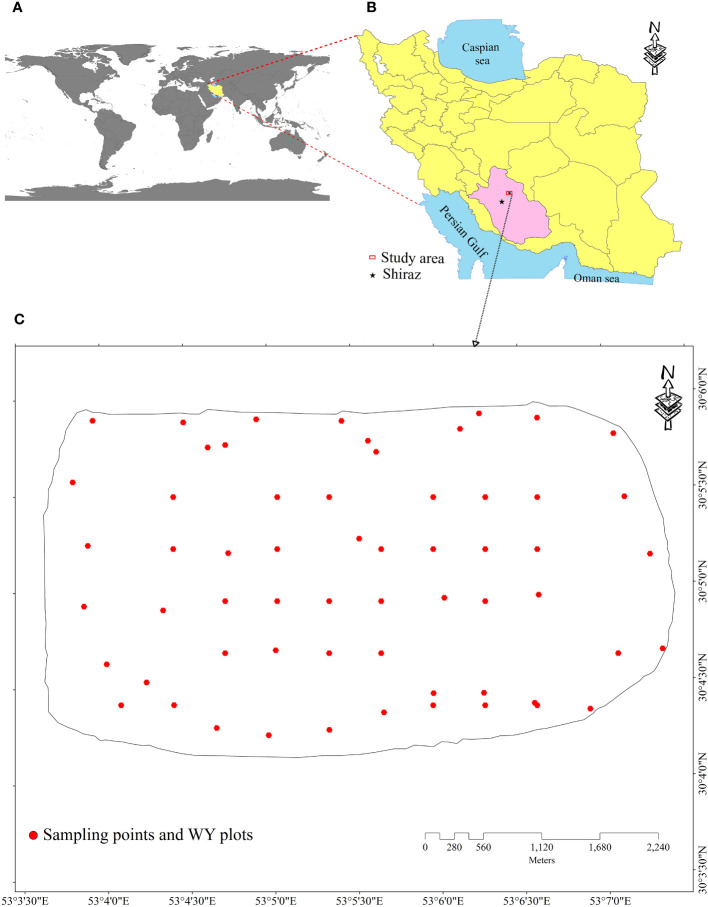
**(A)** Worldwide, **(B)** Iran country, and **(C)** boundary of the study area and sampling soil–wheat yield plots 1 m × 1 m.

The study area covers 1200 ha in the Pasargad plain, located in Fars province in the southwest of Iran (**Figure 1**). Based on climatology data from the closest meteorological station, the area is classified as semiarid with an average rainfall of 350 mm and temperature of 12.5°C. The coldest month is January, and the hottest is July. The study area is situated on the Piedmont and plain landscape with an average slope gradient of 3% and an altitude range of 1747–1780 m. a. s. l. The soils in the study area are classified according to the U.S. soil classification system. They fall under the order of Inceptisols, specifically the Typic Haplocambids and Typic Haplocalcids subgroups (Soil Survey Staff, 2022). The Pasargad plain is a key agricultural zone in Iran that has been under continuous cultivation and exploitation for a long time. The dominant land use in the area is irrigated agriculture, with wheat being the main crop in the crop pattern schedule (Jahandideh Mahjenabadi et al., 2022). So, according to this background, quantifying the production gaps of lands seems to be a very important plan for optimizing agricultural management and future land use planning.

### 2.2 Research workflow

**Figure 2:**
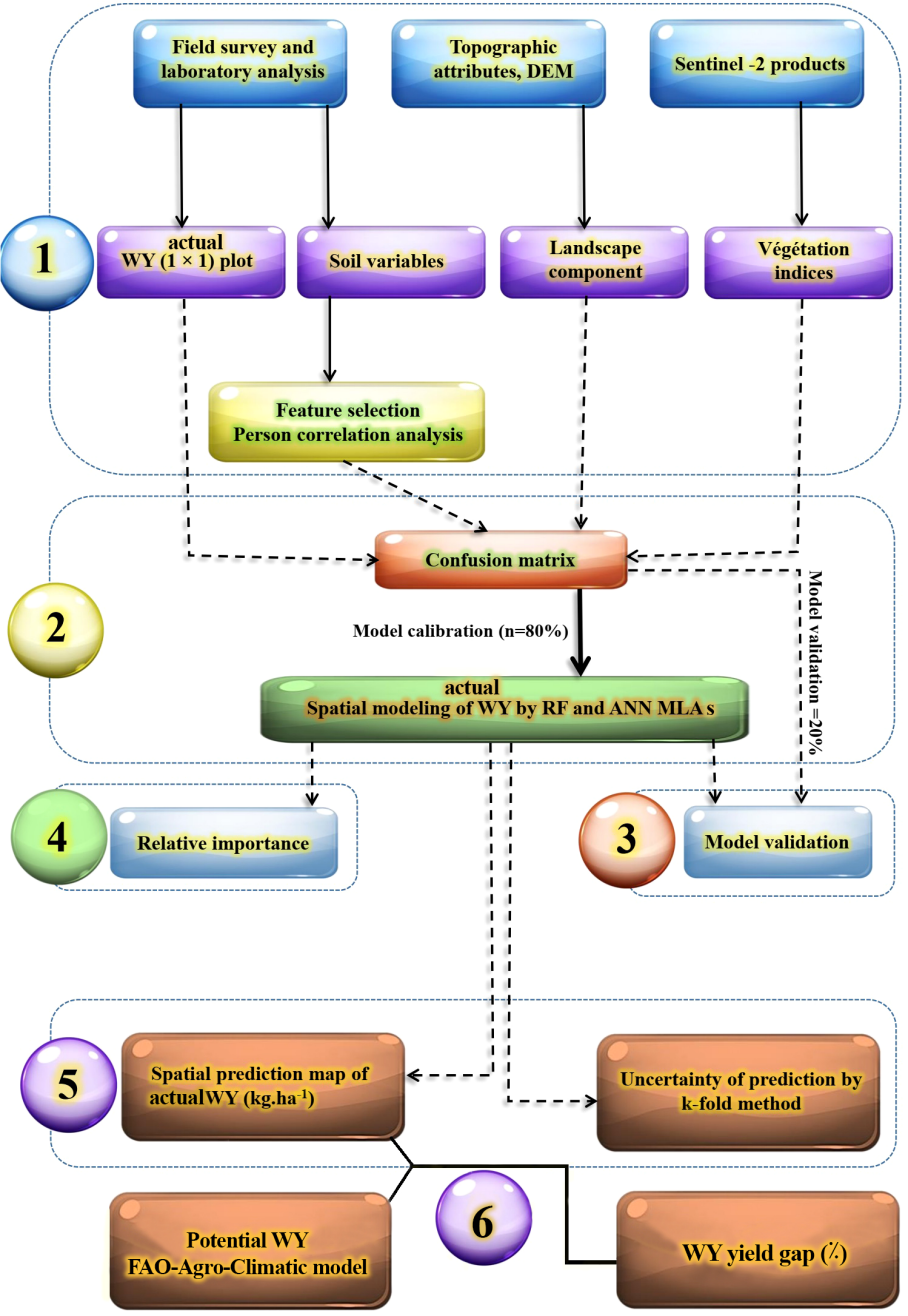
The research work flow of modeling WY (kg ha^−1^).

This research was designed in five main steps, which were carried out in the following order: (1) field survey: soil sampling was conducted from the surface layer (0 cm–30 cm) and WY plots were established at 1 m × 1 m intervals, laboratory analysis was performed to determine physio-chemical properties, and WY (kg ha^−1^) was calculated. In parallel, environmental covariates such as topographic attributes were extracted from DEM and RS data from the Sentinel-2 product. The most important soil variables were selected using Pearson correlation analysis, (2) confusion matrix: the dataset was randomly split into calibration (80%) and validation (20%) sets, and WY was spatially modeled using RF and ANN MLA, (3) validation of MLA performance, (4) determination of the relative importance (RI) of soil and environmental covariates, and (5) preparation of spatial prediction and its uncertainty map using the best ML model and k-fold cross-validation method, respectively (**Figure 2**).

### 2.3 Soil sampling and laboratory analysis

The soil survey and sampling were done at 60 spatial locations in the study area (**Figure 1C**). All soil samples were gathered from 0-cm to 30-cm depth based on a semi-regular method with an average of 500-m interval from 5 May to 15 May 2019. Along with soil sampling, the actual WY (kg ha^−1^) was recorded by using a 1 m × 1 m plot (**Figure 1C**) at four repeats to gather a sample with a representative of WY at each point (same location as the sampling points). Afterward, the soil samples were transferred to the laboratory and were air dried, passed through a 2-mm sieve, and then physical and chemical properties were determinedusing the standard method. The measured soil properties include the soil texture component, that is, sand, silt, and clay (Gee and Bauder, 1986), SOC (Walkley and Black, 1934), TN (Page et al., 1982), cation exchange capacity (CEC) (Sumner and Miller, 1996), pH, and electrical conductivity (EC).

### 2.4 Environmental covariates

The WY predictor variables are composed of three sources: topographic attributes, RS indices, and soil variables. The best set of soil variables was selected based on Pearson correlation analysis. Environmental factors were chosen based on expert opinion and literature (Dedeoğlu and Dengiz, 2019; Wang et al., 2020). For more information, **Table 1** presents the list of soil and environmental covariates used to predict WY. The RS covariates were SAVI (soil-adjusted vegetation index), WDVI, and wetness, which were prepared from the band ratio of sentinel-2 images in SNAP software version 9.0. Additionally, topographic attributes such as texture, convexity, elevation, and CHN were included. These attributes were extracted from the digital elevation model (DEM) in SAGA GIS software version 4.7.

**Table 1:** List of environmental covariates were applied for predicting WY.

Parameters	Symbol	Description	Source
Soil organic carbon	SOC (%)	Soil organic carbon content	Lab analysis
Total nitrogen	TN (%)	Soil total nitrogen content	Lab analysis
Soil-adjusted vegetation index	SAVI	$\left(\text{NIR}-\text{R})\;/\;(\text{NIR}+\text{R}){\;}^{*}\;(1\;+\text{ S}\right)$	Sentinel-2 images
Weighted difference vegetation index	WDVI	WDVI=NIR-(g×R)	Sentinel-2 images
Normalized difference vegetation index	NDVI	NIR−RED/NIR+RED	Sentinel-2 images
Wetness index	Wetness	$\begin{array}{l} 0.0315\;\left(Blue\right)\;+\;0.2021\left(Green\right)\;-\;0.3102\;\left(\mathrm{Red}\right)\;+\;0.1594\;\left(NIR\right)\;-\\ \;0.6806\left(SWIR1\right)\;-\;0.6109\;\left(SWIR2\right) \end{array}$	Sentinel-2 images
Terrain surface texture	Texture	The variations in elevation and roughness of the terrain surface	Digital elevation model
Terrain surface convexity	Convexity	Is a measure of the convexity or concavity degree of a terrain surface	Digital elevation model
Elevation	Elevation (m)	Elevation from sea level	Digital elevation model
Channel network base level	CHN (m)	Difference between the DEM and a surface interpolated from the channel	Digital elevation model

The abbreviations of all covariates are describe in this table.

### 2.5 Machine learning algorithms

The prediction of irrigation WY was accomplished using ANN and RF algorithms, along with auxiliary variables such as topographic attributes, remotely sensed indices, and soil variables. We chose these algorithms due to their success in digital soil mapping, as demonstrated in previous studies (Saeed et al., 2017; Rostaminia et al., 2021; Rezaei et al., 2023). More detailed information about the performance of utilized ML algorithms is given below.

#### 2.5.1 Random forest

RF is a popular algorithm for digital mapping, because it can handle high-dimensional data, nonlinear relationships, and interactions between features. It is also robust to over-fitting and missing values, making it suitable for noisy and incomplete data (Breiman, 2001). The RF utilizes a collection of decision trees, where each tree is created using a randomly selected subset of the training data and variables. Decision trees were introduced by (Breiman et al., 1984) and consist of binary bifurcations that recursively split the training data by selecting the variable and threshold at each split, which creates two subsets with the highest degree of homogeneity possible. One of the advantages of RF models is that they select both the training data and candidate variables for each split of each tree, which reduces overfitting and improves prediction accuracy. Additionally, RFs provide information about the importance of each variable used in the prediction. Here, the RF is impeded by the “random forest” package, and it was tuned by mtry and ntree hyperparameters by the “caret” package for predicting WY.

#### 2.5.2 Artificial neural network

ANNs are dynamic computational networks that are capable of describing intricate nonlinear relationships among related variables (Ripley, 1996; Were et al., 2015). ANNs are essentially a collection of functions that can be used to fit algorithms without making any assumptions about the distribution of errors (Gahegan, 2003). This makes ANNs a highly flexible and powerful technique, offering the potential advantage of abstraction when applied to large-scale domains. When running an ANNs model, two parameters, size and weight decay coefficient, must be optimized. Size refers to the number of neurons in the hidden layer, and the weight decay coefficient is a tuning parameter to prevent over-fitting of the model so that the weights are multiplied by a coefficient less than 1 at each update. This prevents the weights from growing too large, which usually changes logarithmically. In this study, the values of 0.1, 0.01, and 0.001 were evaluated by the caret package and ultimately optimized with a weight coefficient of 0.1 and 5 hidden layer neurons.

### 2.6 Model validation and uncertainty analysis

#### 2.6.1 Model validation

To validate the model, a random holdback cross-validation procedure was used by randomly partitioning the dataset into 80% for training and 20% for testing. This allowed us to train our models on a subset of the data while reserving a portion of the data for model evaluation to ensure the robustness of our results. Also, 10-fold cross-validation method with 10 repetitions during the model training process, where the dataset is divided into 10 equal parts, with each part used as the validation set once, while the remaining nine parts are used as the training set. The purpose of this approach is to ensure that the model is trained on a representative sample of the data and to minimize the risk of overfitting. Finally, model hyperparameters were fine-tuned the using grid search and cross-validation techniques to optimize the model’s performance (Kumar, 2018; Meier et al., 2018). For evaluating the model’s accuracy, the coefficient of determination (*R* ^2^), Lin’s concordance correlation coefficient (CCC), root-mean-square error (RMSE), and relative percent difference (RPD) were calculated. When RPD is less than 1.0, the prediction performance is poor; when RPD is between 1.0 and 1.4, the prediction performance is only useful for determining high and low data; when RPD is between 1.4 and 2.0, prediction performance is fair; when the RPD is between 2.0 and 2.50, the results of prediction and applied models are strong, and if RPD is more than 2.5, the prediction performance is excellent (Chang et al., 2001). The accuracy metrics were calculated as follows (Equations 1–4):


(1) $$R^{2}=\frac{\sum_{i=1}^{n}{\left(a_{i-}\bar{b_{i}}\right)}^{2}}{\sum_{i=1}^{n}{\left(b_{i-}\bar{b_{i}}\right)}^{2}}$$

(2) $$RMSE=\sqrt{\frac{1}{n}\sum_{i=1}^{n}{\left(a_{i}-b_{i}\right)}^{2}}$$

(3) $$CCC=\frac{2r\partial_{a}\partial_{b}}{\partial_{a}^{2}+\partial_{b}^{2}+{\left(\bar{a}+\bar{b}\right)}^{2}}$$

(4) RPD=SD/RMSE

where, a_i_ and b_i_ are the observed and predicted values, $\bar{a}$ and $\bar{b}$ , are the average of the observed and predicted values, *r* is the correlation coefficient between the observed and predicted values, and $\partial_{a}$ , and $\partial_{b}$ are the variance of the observed and predicted values. To assess the accuracy of the results and model performances, the Kruskal–Wallis (KW) test was used to identify any statistically significant differences in performance among MLAs (Demir and Citakoglu, 2023; Rezaei et al., 2023).

#### 2.6.2 Uncertainty analysis

There are various approaches for quantifying the uncertainty of model outputs, and one of these methods is the empirical approach that uses residuals between modeled outputs and observed data to quantify the prediction interval. k-fold cross-validation is a statistical technique used to evaluate the performance of a ML model. It involves splitting the data into k subsets, or folds, where k is a positive integer. The model is then trained on k-1 of these folds and evaluated on the remaining fold. This process is repeated k times, with each fold serving as the test set exactly once. The results are averaged across the k iterations to produce a more robust estimate of the model’s performance. k-fold cross-validation is a commonly used method for assessing the uncertainty of prediction maps, as it provides a measure of how well the model generalizes to new data. In this study, uncertainty was estimated using 10-folds (Vanwinckelen and Blockeel, 2012). The implementation of this method involved the use of ML models and coding in the open-source statistical software R.

### 2.7 Attainable potential of wheat and yield gap

Calculating the potential production of crops is of utmost importance in agricultural planning and management. Yield potential is defined as the yield of a cultivar when grown in environments to which it is adapted, with non-limiting nutrients and water supplyand pests, diseases, weeds, lodging, and other stresses effectively controlled (Evans and Fischer, 1999). By determining the potential production of different crops, farmers can allocate their resources, such as land, water, and fertilizers, more efficiently. This helps maximize yields and minimize waste (Taghizadeh-Mehrjardi et al., 2020). The estimation of the potential yield of irrigated wheat is a crucial aspect of crop management. The FAO-agro-climatic model is a widely used approach that incorporates genetic potential plant and climate data, including radiation, temperature, and land potential, to determine the expected production. Researchers can refer to Sys et al. (1991); Mousavi et al. (2017), and Taghizadeh-Mehrjardi et al. (2020) for a more comprehensive understanding of the calculation process involved in determining the potential yield of irrigated wheat using the FAO model. Furthermore, for quantifying the yield gap the relation proposed by Fischer et al. (2014) yield gaps was applied and the value is expressed as a percentage of actual and potential yield as (Equation 5):


(5) $$Yield\;Gap=1-\frac{actual\;production}{Potential\;production}\times 100$$

## 3 Results

### 3.1 Summary statistical of wheat yield

The summary statistics of WY and soil-environmental covariates are presented in **Table 2**. The minimum and maximum WY are varied from 2500 kg ha^−1^ to 7430 kg ha^−1^, with a mean value of 4937 kg ha^−1^. From the variability of WY and soil-environmental covariates according to their CV (%), the results demonstrated that SOC, TN, elevation, and CHN are in the low variability class, WY, WDVI, wetness, and convexity are in the moderate, and SAVI and NDVI are in the high-variability class according to the category defined by Wilding (1985).

**Table 2:** Summary statistics of WY and environmental covariates at 60 point and plot observation.

Parameters	Unit	Min	Max	Mean	Median	SD	ABS CV (%)
WY	kg ha^−1^	2500	7430	4937	4750	1117	22.6
SOC	%	0.79	1.38	1.08	1.07	0.12	11.0
TN	%	0.08	0.14	0.11	0.11	0.01	11.1
SAVI	–	−0.04	1.07	0.51	0.36	0.33	65.0
WDVI	–	1036	4666	2688	2495	762	28.4
NDVI	–	−0.03	0.71	0.34	0.24	0.22	65.0
Elevation	m	1754	1770	1762	1761	4.12	0.23
CHN	m	1753	1767	1760	1760	3.40	0.19
Wetness	–	−4453	−1509	−3296	−3354	547	16.6
Texture	–	0.00	45.8	7.48	2.44	10.3	137
Convexity	–	9.82	54.2	35.16	36.03	10.05	28.6

Min, minimum; Max, maximum; SD, standard deviation; ABS CV, absolute coefficient of determination; WY, wheat yield; SOC, soil organic carbon; TN, total nitrogen; SAVI, soil-adjusted vegetation index; WDVI, weighted difference vegetation index; NDVI, normalized difference vegetation index; CHN, channel network base level; Wetness, wetness index; Texture: terrain surface texture; Convexity, terrain surface convexity.

### 3.2 Correlation analysis of WYS and soil variables

**Figure 3:**
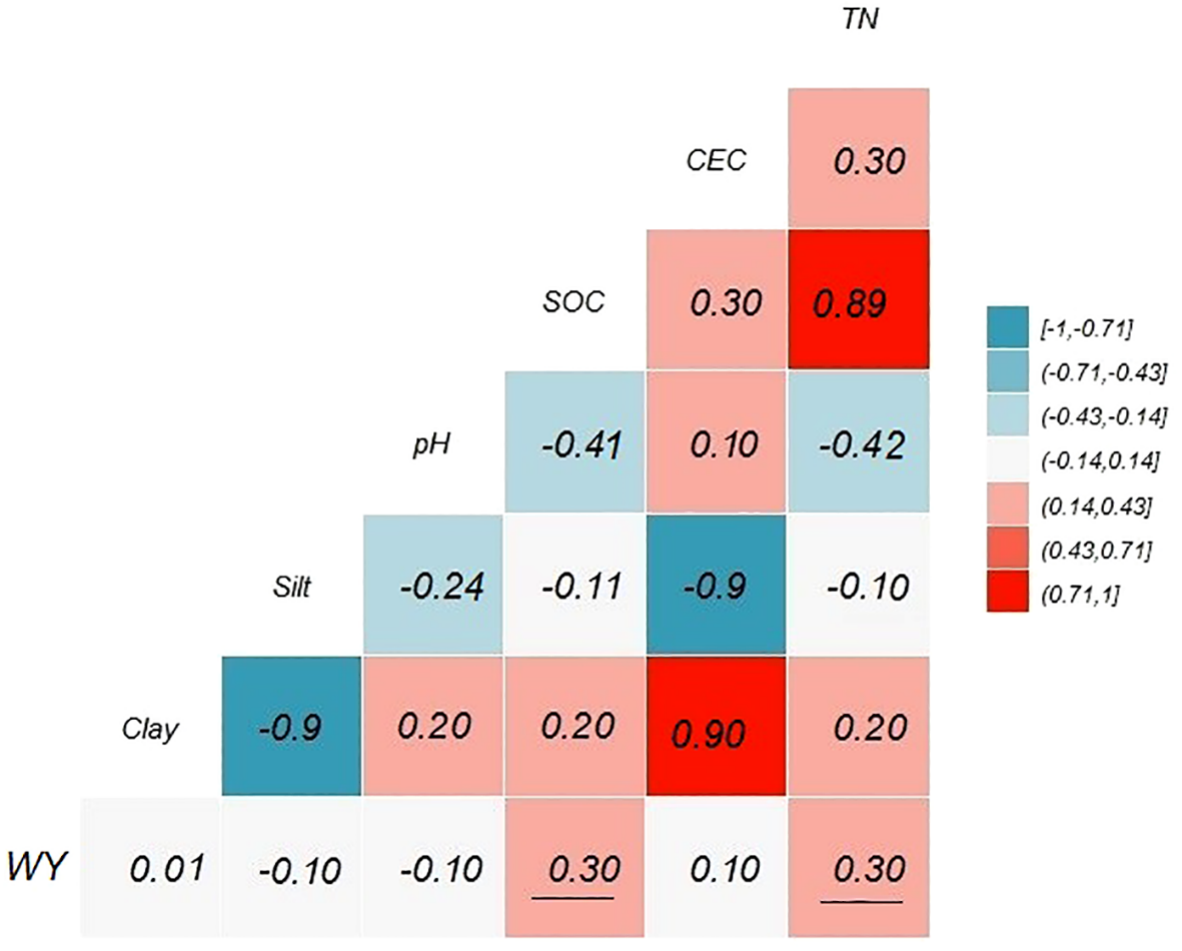
Pearson correlation coefficient test between WY (kg ha^−1^) and soil physico-chemical properties. Underlined correlation values between WY and soil variables are significantly different from zero (*p* = 0.05).

The results of relationship between soil variables and WY are presented in **Figure 3**. According to the correlation results, only soil organic carbon (SOC) (*r* = 0.30) and TN (*r* = 0.30) had a positive and significant correlation with WY. Other soil variables (CCE, pH, Silt, Clay, and Sand) did not show a significant correlation in this analysis.

### 3.3 Comparison of machine learning models

The ability of two MLA (ANNs and RF) to predict actual WY in the study area was validated based on fivefold with 10 times cross-validation. Results for the *R* ^2^, CCC, RMSE, and RPD for each MLA are presented in **Table 3**. The *R* ^2^, RMSE, and RPD for WY using the ANNs algorithm (0.75, 0.80, 400, and 2.79, respectively) were better than the RF algorithm (0.68, 0.72, 500, and 2.20, respectively). Therefore, both algorithms performed well in predicting actual WY, while from a statistical point of view, the ANNs algorithm performed better than RF. The results of the KW test (*p* = 0.05) indicated that the models’ predictions were robust with minimal errors, and there was no statistically significant difference in the prediction performance between the ANN and RF models (**Table 4**).

**Table 3:** Model prediction performance statistics for RF and ANN models applied to actual WY prediction.

Statistical indices	ML models	
	RF	ANN
R2	0.68	0.75
CCC	0.72	0.80
RMSE (kg ha^−1^)	500	400
RPD	2.20	2.79

**Table 4:** The results of the Kruskal–Wallis (KW) test of accuracy of predictions.

ML models	*P*-value	Critical value	H_o_^*^
ANN-RF	0.62	0.05	Rejected

H0*: There is no statistically significant difference in the prediction performance between MLAs.

### 3.4 Relative importance of predictors

**Figure 4:**
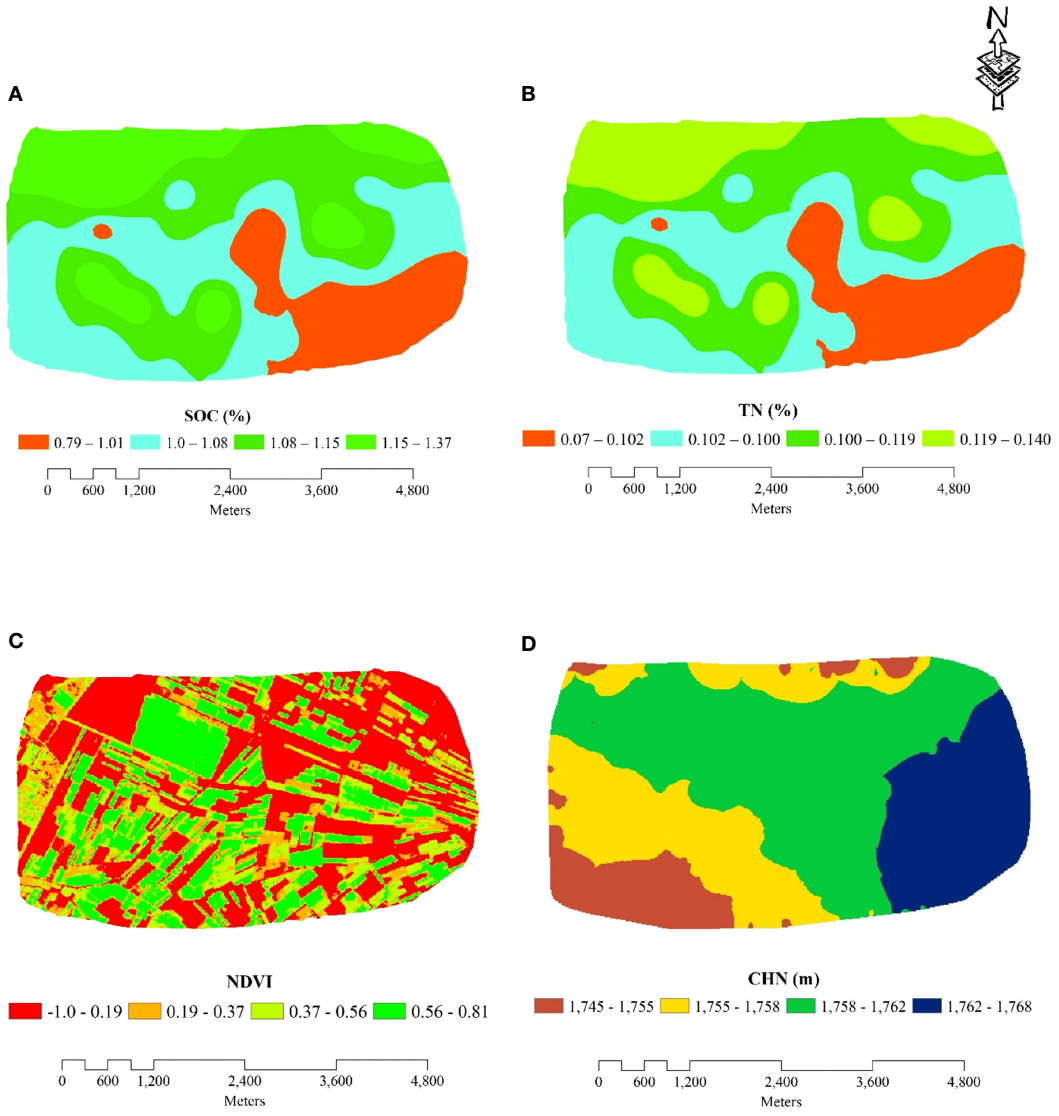
Four important covariates based on RI analysis. **(A)** SOC (soil organic carbon), **(B)** TN (total nitrogen), **(C)** NDVI (normalized difference vegetation index), **(D)** CHN (channel network base level).

**Figure 5:**
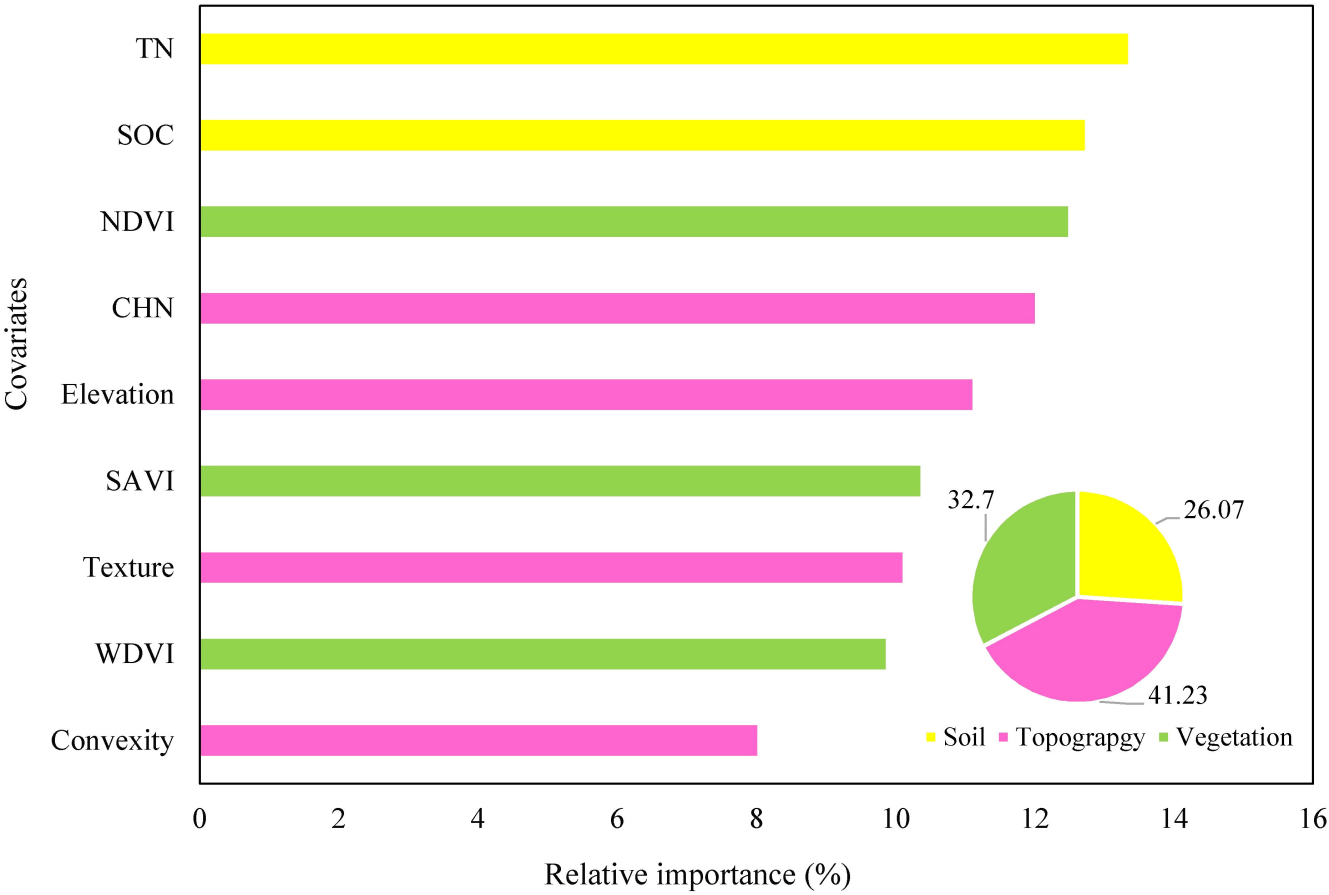
The RI (%) of soil, topography and vegetation indices for predicting actual WY in the study area. WY, wheat yield; SOC, soil organic carbon; TN, total nitrogen; SAVI, soil-adjusted vegetation index; WDVI, weighted difference vegetation index; NDVI, normalized difference vegetation index; CHN, channel network base level; wetness, wetness index; texture, terrain surface texture; convexity, terrain surface convexity.

Given the fact that the ANN model outperformed in predicting actual WY, the RI results were discussed based on the output of ANNs. In total, nine soil and environmental covariates were utilized for modeling and generating a prediction map of actual WY, as demonstrated in **Table 1**. For illustrative purposes, the maps corresponding to the top four covariates are presented in **Figure 4**. The results of the RI analysis indicated that SOC, TN, NDVI, and CHN were found as the most influential covariates, and account for 13.5%, 13%, 12.5%, and 12% of the total variance of WY, respectively (**Figure 5**). Furthermore, the combination of these top four covariates covers more than 50% of WY. It is worth noting that SOC and TN are related to soil properties (**Figures 4A, B**), NDVI is associated with vegetation indices (**Figure 4C**), and CHN serves as a proxy for topography (**Figure 4D**). NDVI was the third important factor in the prediction of WY. Based on the field observations, the WY recording was done among ripening and harvesting dates, so it is revealed that the NDVI can be a powerful vegetation index for predicting/monitoring crop yield in this period of the wheat growth cycle. CHN, as a proxy of topography, was identified as the fourth top important covariate in the prediction of WY by affecting the movement of water across the landscape, which in turn influences soil moisture and nutrient availability for the crops. From the source of utilized covariates, the quantitative results of RI demonstrated that topographic attributes (41.23%) followed by soil variables (32.70%), and vegetation indices (26.07%) had the largest potential for predicting actual WY in the study area.

### 3.5 Spatial prediction, uncertainty map, and wheat yield gap

**Figure 6:**
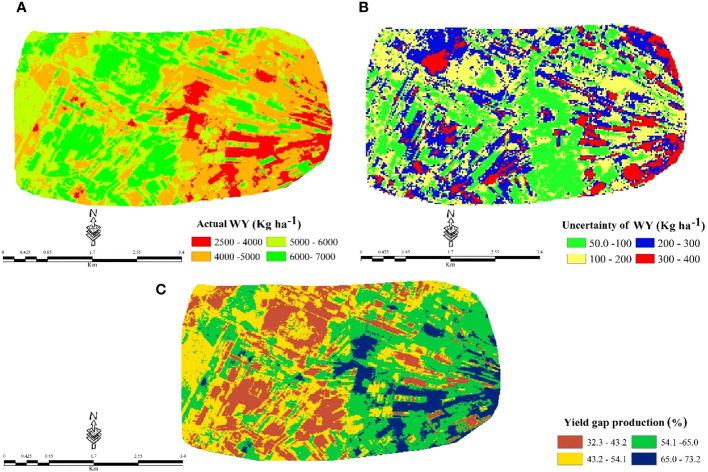
Spatial prediction of **(A)** actual (kg ha^−1^), **(B)** uncertainty (kg ha^−1^), and **(C)** yield gap (%) maps of WY by ANN model.

According to **Figure 6** and previous sections, the prediction map of actual WY was created using the spatial distribution described by ANNs. Based on the prediction map, the minimum and maximum values varied from 2500 (kg ha^−1^) to 7000 (kg ha^−1^) of WY in the area, and the trend of the prediction map also revealed that more than 60% of the study area, mostly in the northern, western, southwest, and part of the central, had the highest actual wheat production in the range of 5000 to 7000 (kg ha^−1^). For instance, the trend of WY prediction maps strongly corresponds to SOC, TN, and then NDVI (**Figures 4**, **5**). The lower actual WY content was mostly observed in the eastern part of the area (**Figure 6A**). These areas are consistent with the CHN pattern shown in **Figure 4D**, where higher values of CHN are related to erosion and the loss of soil nutrients. According to the field observations, the area with a high-actual WY content has better management by the farmer, in addition to fertilizer and soil organic matter.

The uncertainty analysis by the k-fold method showed that the maximum uncertainty of the prediction map [400 (kg ha^−1^)] was very low compared to the mean value [4937 (kg ha^−^)] of the WY map (**Figure 6B**). The identification of the areas with the highest uncertainty, particularly in the east and northeast zones with the lowest WY values, is significant as it draws attention to the need for more focused and effective soil fertility management and crop system strategies in these areas.

The calculated potential WY by the FAO-agro-climatic method (Sys et al., 1991) was equal to 10350 kg ha^−1^. This result is comparable with Zeynadini et al. (2020) findings in the Sepidan plain, adjacent to the study area, with a yield of 9846 kg ha^−1^. This indicates a significant range yield gap of 32.3% to 73.2% between the maximum and minimum of actual WY production according to potential yield content (**Figures 6A, C**). These results justify the variability and potential for improvement in WY production within the study area. So, by identifying the factors that contribute to the variability in WY production and implementing targeted interventions to address these factors, it may be possible to increase WY production and narrow the range of the gap between the limits of actual WY production. Based on the RI analysis (**Figure 5**), it was found that TN and SOC are the most significant factors affecting the prediction of actual yield. Therefore, it appears that a significant portion of the current yield gap can be attributed to soil fertility (i.e., physical, chemical, and biological properties) and water supply. It is acknowledged that little information about soil profiles is available, however, the RI and Pearson correlation analysis illustrated that the most important land suitability assessment indices, for example, salinity, alkalinity, CEC, particle size distribution, and slope gradient, were not limiting factors in the Pasargad plain.

## 4 Discussion

In this study, the importance of accurately predicting actual WY has been underscored for ensuring global food security and fostering sustainable agricultural practices. The primary objective was to discern the key soil and environmental factors influencing WY in a specific segment of irrigated croplands in southwest Iran. This investigation has employed the FAO-agro-climate method in conjunction with machine learning algorithms (MLAs) to achieve a comprehensive understanding of the factors influencing WY in the designated region.

Low to moderate variability in the topographic attributes seems to be more related to the physiography of the study area, with a mean slope gradient close to 3%. For soil properties, similar results were reported by Mosleh et al. (2016) and Mousavi et al. (2022) in areas with similar topographic conditions. High variability of vegetation indices, for example, NDVI and SAVI indicate the wheat heterogenic of cultivation schedule by farmers in the study area.

NDVI and SAVI are commonly used to assess vegetation health and vigor, and their variability can be indicative of differences in crop growth stages, health, or even the implementation of various agricultural practices (Pettorelli et al., 2014). It is possible that farmers in the study area do not follow uniform cultivation practices for wheat. This diversity in cultivation schedules could be influenced by climate variations, individual farming practices, or socio-economic considerations (Laso Bayas et al., 2017). It highlights the complexity of agricultural activities in the region and emphasizes the need for a nuanced understanding of the interactions between topography, soil, and vegetation in the context of agricultural practices. The discussed findings underscore the importance of considering the physiographic characteristics of a region when studying its topography, soil properties, and vegetation dynamics. The observed patterns in the study area suggest a connection between the landscape features and the agricultural practices employed by farmers. This insight contributes to a more comprehensive understanding of the intricate relationships between natural elements and human activities in the context of agricultural landscapes.Correlation analysis showed that an increase in SOC and TN content in the soil can increase actual WY (Grant et al., 2001; Fan et al., 2005). Similar results were observed by Zhang et al. (2009), who indicated that both wheat and corn grain yields are significantly correlated with SOC, TN, and phosphorus. As regards, Kumhálová et al. (2008) observed a positive and significant correlation coefficient among SOC and TN with winter rape. For instance, applying SOC and soil fertilizer (e.g., TN) can lead to an increase in water holding capacity, soil porosity, aggregate stability, and a decrease in soil compaction and surface crusting, which can result in high crop production (Kanchikerimath and Singh, 2001). Therefore, the SOC and TN were known as representatives of soil in the spatial modeling of the actual WY.

The variations in soil physical, chemical, and biological properties are largely influenced by changes in SOC and TN (Monaco et al., 2008), which have a great impact on crop productivity (Sainju et al., 2008). SOC and TN play a crucial role in shaping soil structure and influencing various soil properties. Increased organic matter content resulting from these components enhances soil aggregation and stability, thereby impacting soil texture (Smith et al., 2008). This, in turn, affects important soil functions such as water retention, drainage, and aeration. High levels of SOC contribute to improved soil porosity, enhancing water infiltration, root penetration, and the movement of gases within the soil (Six et al., 2000). Furthermore, SOC and TN act as significant sources of nutrients for plants. Microbial decomposition of organic matter releases essential nutrients such as nitrogen, phosphorus, and sulfur, directly influencing plant growth and soil fertility (Bardgett and van der Putten, 2014). SOC also serves as a buffer for soil pH, with organic acids produced during decomposition mitigating changes in pH and contributing to a more stable and favorable pH range for plant growth (Richter and Markewitz, 1995). Additionally, SOC is a substrate for soil microorganisms, and the availability of SOC and TN influences the diversity and activity of soil microbes (Fierer et al., 2012; Khoshru et al., 2020a). A rich microbial community supported by organic matter enhances soil biological activity, promoting symbiotic relationships with plants, disease suppression, and overall ecosystem resilience (Ens et al., 2009; Khoshru et al., 2020b). The water-holding capacity of soil is improved by SOC acting as a sponge, aiding in water retention during dry periods and providing a steady water supply to plants (Nimmo, 2004). Moreover, SOC contributes to the binding agents (glues) that hold soil particles together in aggregates, thereby enhancing soil structure and stability, reducing erosion, and promoting better water infiltration (Tisdall and Oades, 1982). The C:N ratio in organic matter is a crucial factor affecting the rate of decomposition. A balanced C:N ratio promotes efficient decomposition, ensuring a steady release of nutrients without causing nitrogen imbalances (Moorhead and Sinsabaugh, 2006). Understanding the intricate interplay between SOC, TN, and various soil properties is essential for sustainable soil management. Practices that enhance organic matter content, such as cover cropping, crop residue incorporation, and organic amendments, can positively influence soil physical, chemical, and biological attributes, contributing to improved soil health and agricultural productivity (Paul and Clark, 1996).

Upon further analysis and consideration, we hypothesize that the absence of significant correlations for some soil variables (CCE, pH, Silt, Clay, and Sand) could be attributed to their indirect effects on the measured outcome. Soil processes are complex and interconnected, and certain soil variables may influence the outcome indirectly through their impact on other mediating factors. Soil properties rarely act in isolation. Instead, they often interact with each other and with other environmental factors. For instance, while CCE, pH, and soil texture (silt, clay, and sand) may not directly impact WY, they could influence nutrient availability, water retention, or soil microbial activity, which in turn affect crop growth and productivity (Brady and Weil, 2008). Soil pH, for example, can influence the availability of essential nutrients to plants (Neina, 2019). If the pH is within an optimal range, nutrient uptake may be adequate, even if a direct correlation with pH is not observed. Similarly, CCE can influence nutrient exchange capacity, indirectly affecting nutrient availability. Soil texture, which includes the proportions of sand, silt, and clay, influences water retention. While these variables may not directly correlate with WY, they can indirectly impact crop productivity by influencing water availability to plants. Certain soil properties can influence microbial communities, and these microorganisms play a crucial role in nutrient cycling and organic matter decomposition. The indirect effects of soil microbial activity on nutrient availability can impact crop yield (Fierer et al., 2012). EC is often an indicator of soil salinity. While it may not directly correlate with WY, salinity can have indirect effects on plant water uptake and nutrient absorption, affecting overall crop health and productivity (Munns and Tester, 2008). Soil conditions can vary spatially and temporally. The lack of a direct correlation in a specific study may be influenced by the specific conditions and the timing of data collection. Similarly, (Nabiollahi et al., 2020) observed that the lack of significant correlations for some soil properties (CCE, pH, Silt, and EC) with the measured WY may not directly impact the WY in their study area.

Several studies have highlighted the effectiveness of ANNs in predicting crop yield based on various factors such as weather, soil quality, and management practices. As regards, Miao et al. (2006) emphasized the importance of ANNs in modeling complex nonlinear relationships between input and output variables for crop yield prediction. Similarly, Ayoubi and Sahrawat (2011) and Norouzi et al. (2010) successfully utilized ANNs to predict grain yield based on soil properties collected and analyzed through traditional lab methods in Iran. These findings confirm the capability of ANNs in predicting crop yield, which is particularly significant given that crop yield depends on a variety of factors. Kadir et al. (2014) demonstrated the promising potential of ANNs predict WY, suggesting that this approach can be applied to other crops as well. Other studies, such as Irmak et al. (2006), Drummond et al. (2003), and Liu et al. (2001), also reported successful outcomes in using ANNs for data mining, crop yield prediction based on soil properties, and determining target corn yields, respectively. However, Taghizadeh-Mehrjardi et al. (2020) found that RF outperformed support vector machines in land suitability prediction for wheat and barley yields. Additionally, Jahandideh Mahjenabadi et al. (2022) found that the RF model was effective in predicting winter WY when considering only soil biological properties as predictors.

Jahandideh Mahjenabadi et al. (2022) conducted a comprehensive study in the Southwest of Iran, employing ML algorithms to predict the spatial distribution of soil biological properties and WY. The investigation involved collecting topsoil samples from 60 locations, recording wheat grain yield at each site, and measuring various soil properties, including urease, alkaline phosphatase, basal respiration, microbial biomass carbon, SOC, MBC : SOC ratio, and metabolic quotient. They utilized the RF model in the initial phase to predict soil biological properties. They were employedsix ML algorithms to model wheat grain yield. These models were optimized and evaluated using 10-fold cross-validation with the Caret package. Results revealed varying prediction accuracies among soil biological properties, with qCO_2_ demonstrating the highest accuracy (*R* ^2^ adj = 0.80) and BR the lowest (*R* ^2^ adj = 0.23). Soil covariates played a significant role in modeling urease (Ur), qCO_2_, microbial biomass carbon (MBC), and the MBC : SOC ratio. Specific environmental predictors, such as bands 6 and Chanel Network Base Level, were identified as crucial for alkaline phosphatase (AP) and basal respiration (BR), respectively. Regarding wheat grain yield prediction, both Stochastic Gradient Boosting (SGB) and RF models outperformed other algorithms, achieving impressive *R* ^2^ adj values of 0.89 and 0.88, respectively. The study underscored the significance of urease (Ur) and alkaline phosphatase in predicting wheat grain yield and elucidating its spatial variability.

The emphasis on soil biological properties suggests that indicators of soil health, such as microbial biomass, enzymatic activity, and microbial diversity, are integral to understanding and predicting winter WY. Healthy soil biology can contribute to nutrient availability, organic matter decomposition, and other processes that impact crop growth (de Faria et al., 2021). Soil microorganisms play vital roles in nutrient cycling, disease suppression, and overall plant health. The finding suggests that the activity and diversity of these microorganisms, as reflected in soil biological properties, are important factors influencing WY (Philippot et al., 2013).

The effectiveness of the RF model implies that the relationships between soil biological properties and winter WY are likely complex and nonlinear. Traditional linear models may struggle to capture these intricate relationships, and ensemble methods like RF are well suited for handling such complexities (Breiman, 2001). It is crucial to assess the model’s performance across different datasets or geographic locations to validate its generalizability. This would help determine whether the observed effectiveness of the RF model holds true under various conditions.The average wheat production in the country has values of around 3000 (kg ha−^1^) (Jahandideh Mahjenabadi et al., 2022). In contrast with country records, Khosravi et al. (2023) in the vicinity of the study area, observed an average yield of 6750 (kg ha−^1^) for winter wheat. It can be inferred that the spatial prediction of actual WY is highly dependent on a combination of various factors in the surrounding environment, such as soil and vegetation. Similarly, these results correspond to what Monaco et al. (2008) indicated in this context.

The contrast between the average wheat production values reported is indicative of significant spatial variability in wheat production. This discrepancy underscores the influence of local factors on crop yield and the importance of considering the specific conditions of a region when assessing agricultural productivity. This spatial variability can be attributed to diverse environmental conditions, including variations in soil properties, climate, and land management practices (Lobell et al., 2009). The inference that spatial prediction of actual WY is highly dependent on a combination of various factors in the surrounding environment aligns with the understanding that local conditions play a crucial role in determining crop outcomes. Factors such as soil quality, water availability, temperature, and topography can vary significantly from one location to another, contributing to differences in crop performance (Lobell et al., 2009). Tailoring agricultural practices to the specific characteristics of each location can optimize productivity and resource use efficiency. Precision agriculture, where technologies such as remote sensing, GPS, and data analytics are employed to customize farming practices at a finer spatial scale allows farmers to adapt their strategies based on the specific conditions of different field areas (Tao et al., 2021).

Ren et al. (2021) confirmed in their crop modeling study that winter WY can be accurately estimated before harvesting dates, provided that adequate NDVI data are available. This capability has also been demonstrated by Boori et al. (2023) for rice. The NDVI is known for its ability to indicate green biomass or nitrogen content, particularly in the plant canopy. In the case of wheat, studies have found a strong correlation between the peak of NDVI and yield, which was closely related to the crop reproductive stage (Skakun et al., 2017). Similarly, a study conducted by Panek and Gozdowski (2020) investigated the relationship between NDVI and cereal grain yield, finding a strong correlation between NDVI and grain yield, specifically during the period from March to May (similar to our research). Even a slight increase in NDVI (e.g., 0.1) during the spring season resulted in a substantial rise in grain yield, ranging from approximately 1.1 to 2.6 tons per ha. Topography attributes also impact crop production by affecting microclimate and related soil factors such as temperature, which in turn influence germination, tiller production, and overall crop growth (Godwin and Miller, 2003). Topographical data in combination with soil information are useful for explaining yield variability on an agricultural field scale (Kravchenko and Bullock, 2000). Moreover, terrain attributes such as elevation, plan, and profile curvatures, and relative slope position influence soil properties and classification (Beaudette et al., 2013). The spatial variability of texture and other soil properties at the field scale concerning terrain attributes links the nature of the variability with water movement and nutrient dynamics within the soil (Bobryk et al., 2016; Kokulan et al., 2018).

Land managers and farmers may need to pay closer attention to the factors that are contributing to the low WY values and high uncertainty levels, such as soil type, land use, and management practices. Baltensweiler et al. (2021) believed that the effect of the number of observations on model uncertainty was significant. Therefore, additional field observations/records and monitoring systems are necessary to reduce uncertainty levels in large-scale areas. Our findings are supported by Mueller et al. (2012) and Sinclair and Rufty (2012), who concluded that nutrient and water management, are crucial in closing the yield gap. Therefore, fertilizer use, irrigation, and climate significantly affect yield variability, and crop yield increases are more closely associated with nitrogen and water management than plant genetics. Actual yield is sensitive to growth-reducing factors such as disease, pests, and weeds in humid areas (Grassini et al., 2015). Furthermore, Shimoda et al. (2022) demonstrated that the yield gap is caused by humidity damage and can be reduced through breeding improvement.

## 5 Conclusion

The study was conducted with the global aim of preparing a spatial prediction maps of the actual WY and yield gap and identifying the most important factors in the study area.

The study emphasizes the significance of considering multiple covariates when predicting WY. The findings suggest that SOC, TN, NDVI, and CHN are the most important predictors for actual WY and can be used to enhance the precision of spatial predictions. In terms of RI, the quantitative results showed that topographic attributes had the greatest potential in actual WY prediction, followed by soil variables and vegetation indices.

The performance of MLA showed that the ANNs algorithm outperformed the RF algorithm with higher *R* ^2^, CCC, and RPD values and lower RMSE values, although both considered algorithms had acceptable accuracy in digital mapping of actual WY. The results of the uncertainty analysis also confirmed the high potential of the applied methodology for mapping crop production in other parts of the country and areas with similar environmental conditions.

The prediction map of actual WY revealed that more than 60% of the study area, mostly located in the northern, western, southwest, and part of the central regions, had the highest actual wheat production in the range of 5000 to 7000 (kg ha^−1^), which is higher than the average wheat production in the country and vicinity of the region with values around 3000 and 6750 kg ha^−1^, respectively. We showed that there is a high yield gap between the potential yield production and the actual WY, particularly in areas with low actual yield.

It is recommended that management focus their efforts on these areas to decrease the yield gap and increase farmer income. By mitigating the underlying factors that lead to diminished crop yields, including but not limited to soil composition, irrigation techniques, pest control measures, and the careful selection of cultivars that are tolerant to high salinity and drought conditions, there exists a potential path for enhancing the overall efficiency of wheat cultivation and augmenting the economic returns reaped by farmers. However, this research has provided valuable insights into predicting WY using a ML and DSM framework. The results can be utilized by stakeholders and land managers to plan and increase the productivity of WY in areas with low actual WY and a high production gap.

## Data availability statement

The raw data supporting the conclusions of this article will be made available by the authors, without undue reservation.

## Author contributions

SRM: Writing – original draft, Writing – review & editing, Methodology, Software. VAJM: Writing – original draft, Writing – review & editing, Conceptualization, Funding acquisition, Investigation. BK: Writing – original draft, Writing – review & editing. MR: Conceptualization, Validation, Writing – original draft, Writing – review & editing.
